# Association of Trainee Participation in Colonoscopy Procedures With Quality Metrics

**DOI:** 10.1001/jamanetworkopen.2022.29538

**Published:** 2022-08-31

**Authors:** Michael Sey, Sarah Cocco, Cassandra McDonald, Zaid Hindi, Hasibur Rahman, Debarati Chakraborty, Karissa French, Mohammed Alsager, Omar Siddiqi, Marc-Andre Blier, Bharat Markandey, Sarah Al Obaid, Anthony Wong, Victoria Siebring, Mayur Brahmania, Jamie Gregor, Nitin Khanna, Michael Ott, Karim Qumosani, Aze Wilson, Leonardo Guizzetti, Brian Yan, Vipul Jairath

**Affiliations:** 1 Division of Gastroenterology, Western University, London, Ontario, Canada; 2 Lawson Health Research Institute, Western University, London, Ontario, Canada; 3 South West Ontario Regional Cancer Program, Ontario Health, London, Ontario, Canada; 4 Schulich School of Medicine & Dentistry, Western University, London, Ontario, Canada; 5 Department of Medicine, Western University, London, Ontario, Canada; 6 Department of Pathology, Western University, London, Ontario, Canada; 7 The Royal College of Surgeons in Ireland, Medical University of Bahrain, Adliya, Bahrain; 8 Division of Gastroenterology, Queen’s University, Kingston, Ontario, Canada; 9 Department of Surgery, Western University, London, Ontario, Canada; 10 Division of Clinical Pharmacology, Western University, London, Ontario, Canada; 11 Department of Physiology and Pharmacology, Western University, London, Ontario, Canada; 12 Independent researcher

## Abstract

**Importance:**

Trainees routinely participate in colonoscopy procedures, yet whether their involvement is positively or negatively associated with procedural quality is unknown because prior studies involved small number of trainees and/or supervisors, lacked generalizability, and/or failed to adjust for potential confounders.

**Objective:**

To assess the association between trainee participation and colonoscopy quality metrics.

**Design, Setting, and Participants:**

This multicenter population-based cohort study was conducted at 21 academic and community hospitals between April 1, 2017, and October 31, 2018, among consecutive adult patients undergoing colonoscopy. Procedures performed by endoscopists who did not supervise trainees were excluded. Statistical analysis was performed from April 3, 2017, to October 31, 2018.

**Exposure:**

Participation by a trainee, defined as a resident or fellow enrolled in a gastroenterology or general surgery training program.

**Main Outcomes and Measures:**

The primary outcome was the adenoma detection rate (ADR), and secondary outcomes were sessile serrated polyp detection rate (ssPDR), polyp detection rate (PDR), cecal intubation rate (CIR), and perforation rate.

**Results:**

A total of 35 499 colonoscopies (18 989 women [53.5%]; mean [SD] patient age, 60.0 [14.1] years) were performed by 71 physicians (mean [SD] time in practice, 14.0 [9.3] years); 5941 colonoscopies (16.7%) involved trainees. There were no significant differences in the ADR (26.4% vs 27.3%; *P* = .19), CIR (96.7% vs 97.2%; *P* = .07), and perforation rate (0.05% vs 0.06%; *P* = .82) when trainees participated vs when they did not participate, whereas the the ssPDR (4.4% vs 5.2%; *P* = .009) and PDR (39.2% vs 42.0%; *P* < .001) were significantly lower when trainees participated vs when they did not. After adjustment for potential confounders, the ADR (risk ratio [RR], 0.97; 95% CI, 0.91-1.03; *P* = .30), PDR (RR, 0.98; 95% CI, 0.93-1.04; *P* = .47), and CIR (RR, 0.93; 95% CI, 0.78-1.10; *P* = .38) were not associated with trainee participation, although the ssPDR remained significantly lower (RR, 0.79; 95% CI, 0.64-0.98; *P* = .03).

**Conclusions and Relevance:**

This study suggests that trainee involvement during colonoscopy was associated with reduced ssPDR but not other colonoscopy outcome measures. Extra care should be exercised when examining the right colon when trainees are involved.

## Key Points

**Question:**

Is trainee participation during colonoscopy procedures associated with quality metrics?

**Findings:**

This cohort study found that trainee involvement was associated with a lower sessile serrated polyp detection rate, but not adenoma detection rate, polyp detection rate, or cecal intubation rate, after adjusting for potential patient, endoscopist, and procedural confounders.

**Meaning:**

The results of this study suggest that extra care should be exercised examining the right colon when trainees are involved in colonoscopy procedures.

## Teaser

This cohort study assesses the association between trainee participation and colonoscopy quality metrics of adenoma detection rate, sessile serrated polyp detection rate, polyp detection rate, cecal intubation rate, and perforation rate.

## Introduction

During the past decade, there has been a renewed focus on the quality of colonoscopy procedures worldwide, resulting in the establishment of quality assurance benchmarks that correlate directly with clinical outcomes.^1,2,3,4,5^ Of these metrics, the adenoma detection rate (ADR) has become the most important, owing to its direct correlation with postcolonoscopy risk of colon cancer. In a landmark study involving 45 026 patients and 186 endoscopists, Kaminski et al^6^ found that patients of endoscopists with ADRs less than 11% were more likely to develop an interval colon cancer than patients of endoscopists with ADRs of 20% or more (hazard ratio, 10.94; 95% CI, 1.37-87.01). Similarly, Corley et al^7^ studied 314 872 colonoscopies involving 136 gastroenterologists and found that the ADR was inversely associated with the risk of developing colon cancer after a colonoscopy, with patients of endoscopists in the bottom ADR quintile having the highest interval colon cancer rates compared with patients of endoscopists in the top ADR quintile (hazard ratio, 1.92; 95% CI, 1.45-2.56). As such, there has been great interest in increasing the ADR as a goal of quality improvement.^8,9,10,11^

Although the focus of quality assurance and improvement in colonoscopy procedures has been on the endoscopist, relatively little attention has been paid to whether trainee participation is associated positively or negatively with colonoscopy quality metrics. Unlike board-certified endoscopists, trainees have to balance the need for skill acquisition with the provision of patient care. At times, these competing interests may affect clinical outcomes, as is well reported in the surgical literature.^12,13^ In comparison, there is a paucity of high-quality data on the association between trainee participation and quality outcome metrics in colonoscopy procedures. Therefore, we assessed the association between trainee involvement and colonoscopy quality-related outcomes in a large, population-based cohort study in Ontario, Canada.

## Methods

### Study Design and Population

Consecutive patients who underwent a colonoscopy between April 1, 2017, and October 31, 2018, at 21 academic and community hospitals situated in Southwest Ontario were included in the study. All endoscopy services in the region, with the exception of a single private outpatient endoscopy clinic, were delivered under the auspices of Ontario Health’s South West Local Health Integration Network, a government agency tasked with the coordination of health care in Ontario. The region itself comprises a geographic area of 21 639 km^2^, consists of both rural and urban sites, and is centered on 3 academic hospitals affiliated with Western University, which serves a large referral population of 1.4 million individuals. To form the study cohort, consecutive patients in the region undergoing a colonoscopy for any indication during the study observation period were included. Patients younger than 18 years and endoscopists who performed less than 50 colonoscopies a year or who did not work with trainees were excluded. Approval was obtained from the Western University Health Sciences Research Ethics Board prior to study initiation, and the need for consent was waived because the data had already been collected for quality assurance purposes by the South West Local Health Integration Network. We followed the Strengthening the Reporting of Observational Studies in Epidemiology (STROBE) reporting guideline for observational studies.^14^

### Data Collection

Patient characteristics and endoscopy data were obtained from a custom-made quality assurance endoscopy reporting form that was completed after every procedure. This mandatory reporting form captured the patient’s age, sex, American Society of Anesthesiologists grade, procedure indication, whether the endoscopy was performed as an outpatient or inpatient procedure, and the use of a split-dose bowel preparation. Physician and endoscopy variables were also captured, including procedure date, physician medical license number, hospital name, trainee involvement, bowel preparation quality, sedation used, cecal intubation, polyp detection, and intraprocedural perforation. In Canada, photographic documentation of cecal intubation is performed by taking a picture of the appendiceal orifice or the terminal ileum, often on a physical medium such as photographic paper. Owing to the large number of photographs involved, confirmation of cecal intubation by the study team was not possible. The physician medical license number was cross-referenced with a publicly available provincial physician database^15^ to determine physician specialty and years in practice. An ordinal bowel preparation scale (good, fair, and poor, with poor being considered inadequate) was used because there is no single universally adopted bowel preparation scale used in the province. Delayed perforation was captured using data from the Ontario Health Insurance Plan, the provincially mandated insurer for all Ontarians, and the Canadian Institute for Health Information’s National Ambulatory Care Reporting System and its Discharge Abstract Database, to identify perforation-associated hospitalizations up to 7 days after a colonoscopy. All cases of perforation were manually reviewed. Pathology reports were assessed for any procedure in which a polyp was identified, with the exception of 6 hospitals for which pathology reports were not accessible by the study team (13 686 procedures). These hospitals still contributed data to the analyses, except for those involving ADRs or sessile serrated polyp detection rates (ssPDRs) owing to the lack of pathology data.

The accuracy of the data set was validated by manually reviewing the medical records of 944 of 47 624 randomly selected patients (2.0%) in the cohort for patient characteristics (age and sex) and endoscopy characteristics (cecal intubation, sedation type, polyp detection, and perforation). The patient characteristics were 100% accurate. The endoscopy characteristics were 98.5% accurate for cecal intubation, 92.7% accurate for sedation type, 96.9% accurate for polyp detection, and 100% accurate for perforation.

### Definition of Exposure

The exposure of interest was trainee involvement during colonoscopy. A trainee was defined as a resident or fellow working under the supervision of a board-certified endoscopist. In our region, all trainees learning how to perform colonoscopy actively participate in the procedure and not merely observe. All trainees were affiliated with Western University’s Schulich School of Medicine & Dentistry. Endoscopy training was provided through either the Division of Gastroenterology or the Division of General Surgery. In Canada, gastroenterology trainees complete their internal medicine training during their postgraduate years (PGYs) 1 to 3 and their gastroenterology training during PGYs 4 and 5. General surgery trainees learn how to perform an endoscopy primarily during dedicated endoscopy rotations in PGY 2, although they will further hone their skills between PGYs 3 and 5 during general surgery outpatient rotations. Junior trainees were defined as PGY 4 gastroenterology residents and PGYs 1 and 2 general surgery residents, whereas senior trainees were defined as PGY 5 gastroenterology residents and PGYs 3, 4, and 5 general surgery residents. Advanced fellows with PGYs of 6 or more were board-certified or eligible gastroenterologists or general surgeons pursuing additional training in subspecialized areas, such as hepatology or colorectal surgery. Trainees learned how to perform colonoscopy at both academic and community hospitals, which include both urban and rural settings. Although internists and family physicians also perform colonoscopies in the region, their patients were excluded because they do not supervise trainees.

### Outcome Definition

The primary outcome was the ADR, defined as the proportion of colonoscopies in which a tubular adenoma, tubulovillous adenoma, or villous adenoma were detected. Secondary outcomes included the ssPDR, based on the 2017 US Multi-Society Task Force on Colorectal Cancer definition that excludes hyperplastic polyps^2^; the polyp detection rate (PDR); the cecal intubation rate (CIR); and the perforation rate, which includes both immediate and delayed perforations up to 7 days after a colonoscopy.

### Statistical Analysis

Statistical analysis was performed from April 3, 2017, to October 31, 2018. Descriptive summaries were presented and stratified by trainee participation. Potential differences in baseline characteristics between strata were examined in terms of *t* tests for quantitative variables or likelihood ratio χ^2^ tests for categorical variables. Unadjusted outcome rates among procedures with or without trainees were described as observed. Binary outcomes were reported as risk ratios (RRs), derived from a modified Poisson regression model with cluster-robust variance estimates using the physician as the unit of clustering.^16,17^ This approach relaxes the assumption that the outcome follows a Poisson distribution and directly estimates RRs from binary data. The association of interest was trainee participation status, after adjustments were made for patient characteristics (sex, age, and American Society of Anesthesiologists grade), procedure characteristics (hospital setting, sedation type, split dosing, quality of bowel preparation, indication, and outpatient status), and physician characteristics (years of experience and specialty). Potential effect modification by trainee experience and trainee specialty on the primary outcome was explored using Mantel-Haenszel methods and a test of interaction using the modified Poisson regression method with cluster-robust variance estimates at the trainee level. The level of significance was set at *P* < .05, a priori, and hypothesis testing was 2-sided. A formal sample size calculation, either a priori or post hoc, was not performed owing to the use of a fixed available sample, in keeping with recommendations from the STROBE guidelines.^14^ Statistical analyses were performed using Stata, version 16 (StataCorp LLC).

## Results

### Cohort Characteristics

During the study observation period, a total of 47 624 colonoscopies were performed. Of this initial group of colonoscopies, 368 were excluded owing to being performed by endoscopists who performed less than 50 colonoscopies per year, 456 were excluded owing to missing data on trainee status, 61 were excluded owing to being performed for patients younger than 18 years of age, and 11 240 were excluded owing to being performed by endoscopists who did not supervise trainees, resulting in a final study cohort consisting of 35 499 colonoscopies. The mean (SD) patient age was 60.0 (14.1) years, and 18 989 patients (53.5%) were women (Table 1). The most common indication was investigation of symptoms (19 358 [54.5%]), and most colonoscopies were performed as outpatient procedures (34 011 [95.8%]). There were 24 board-certified gastroenterologists who performed 16 143 colonoscopies (45.5%) and 47 board-certified general surgeons who performed 19 356 colonoscopies (54.5%). The mean (SD) time in practice for the physicians was 14.0 (9.3) years. Overall, 19 522 colonoscopies (55.0%) were performed at academic hospitals.

**Table 1.** Baseline Characteristics of the Study Cohort

Characteristic	Endoscopies, No. (%)			*P* value
	No trainee (n = 29 548)	Trainee (n = 5951)	Total (N = 35 499)	
**Patients**				
Age, mean (SD), y	60.0 (14.0)	60.0 (14.7)	60.0 (14.1)	.94
Sex^a^				
Female	15 891 (53.8)	3098 (52.1)	18 989 (53.5)	.02
Male	13 657 (46.2)	2841 (47.8)	16 508 (46.5)	
ASA grade				
1	7806 (26.4)	1651 (27.7)	9457 (26.6)	<.001
2	14 755 (49.9)	3018 (50.7)	17 773 (50.1)	
3	6552 (22.2)	1154 (19.4)	7706 (21.7)	
4	429 (1.5)	127 (2.1)	556 (1.6)	
5	6 (0.02)	0	6 (0.02)	
Missing	0	1 (0.02)	1 (0.003)	
Primary indication				
Screening	12 385 (41.9)	2359 (39.6)	14 744 (41.5)	<.001
Positive FOBT	1263 (4.3)	128 (2.2)	1391 (3.9)	
Symptomatic	15 895 (53.8)	3463 (58.2)	19 358 (54.5)	
Missing	5 (0.02)	1 (0.02)	6 (0.02)	
Procedure type				
Outpatient	28 816 (97.5)	5195 (87.3)	34 011 (95.8)	<.001
Inpatient	732 (2.5)	756 (12.7)	1488 (4.2)	
Split dosing				
Yes	27 960 (94.7)	5533 (93.1)	33 493 (94.4)	<.001
No	1565 (5.3)	411 (6.9)	1976 (5.6)	
Missing	23 (0.08)	7 (0.1)	30 (0.08)	
Bowel prep quality				
Good	24 758 (83.8)	4835 (81.3)	29 593 (83.4)	<.001
Fair	4029 (13.6)	832 (14.0)	4861 (13.7)	
Poor	750 (2.5)	280 (4.7)	1030 (2.9)	
Missing	11 (0.04)	4 (0.07)	15 (0.04)	
Sedation type				
Standard conscious sedation	19 705/29 376 (67.1)	4894/5923 (82.6)	24 599/35 299 (69.7)	<.001
Deep sedation	9671/29 376 (32.9)	1029/5923 (17.4)	10 700/35 299 (30.3)	
**Physicians**				
Specialty				
Gastroenterology	12 508 (42.3)	3635 (61.1)	16 143 (45.5)	<.001
General surgery	17 040 (57.7)	2316 (38.9)	19 356 (54.5)	
Experience, mean (SD), y	14.1 (9.3)	13.2 (9.5)	14.0 (9.3)	<.001
Academic center	14 281 (48.3)	5241 (88.1)	19 522 (55.0)	<.001

Abbreviations: ASA, American Society of Anesthesiologists; FOBT, fecal occult blood test.

^a^ Two people had “other” recorded as their sex.

### Characteristics of Procedures Involving Trainees

A total of 55 trainees (28 gastroenterology trainees and 27 surgical trainees) performed 5746 colonoscopies (16.7%) during the study observation period. Among the gastroenterology residents, 1037 procedures were performed by junior trainees, 1095 by senior trainees, and 794 by advanced fellows. In the general surgery group, 2162 colonoscopies were performed by junior trainees, 588 by senior trainees, and 70 by advanced fellows (Table 2). Overall, 5241 procedures (91.2%) involving trainees were performed at an academic hospital. Colonoscopies involving trainees were more likely than those not involving trainees to be conducted for diagnostic purposes (58.2% vs 53.8%; *P* < .001), performed as an inpatient procedure (12.7% vs 2.5%; *P* < .001), have poor bowel preparation (4.7% vs 2.5%; *P* < .001), use conscious sedation (82.6% vs 67.1%; *P* < .001), be supervised by a gastroenterologist (61.1% vs 42.3%; *P* < .001), and be performed at an academic center (88.1% vs 48.3%; *P* < .001).

**Table 2.** Characteristics of Trainees

Trainee experience at time of procedure (PGY level)	Specialty, No.		Total No.
	Gastroenterology	Surgery	
1	0	75	75
2	0	2087	2087
3	0	397	397
4	1037	86	1123
5	1095	105	1200
6	648	28	676
7	133	42	175
8	9	0	9
9	4	0	4
Total	2926	2820	5746

Abbreviation: PGY, postgraduate year.

### Outcomes of Procedures Involving Trainees

On univariate analysis, there was no significant difference in the ADR (26.4% vs 27.3%; *P* = .19), CIR (96.7% vs 97.2%; *P* = .07), or perforation rate (0.05% vs 0.06%; *P* = .82) when trainees participated vs when they did not participate, whereas the PDR (39.2% vs 42.0%; *P* < .001) and ssPDR (4.4% vs 5.2%; *P* = .009) were significantly lower when trainees participated vs when they did not (Table 3). On multivariable analysis, there was no association between trainee participation and ADR (RR, 0.97; 95% CI, 0.91-1.03; *P* = .30), PDR (RR, 0.98; 95% CI, 0.93-1.04; *P* = .47), or CIR (RR, 0.93; 95% CI, 0.78-1.10; *P* = .38), although the ssPDR remained significantly lower (RR, 0.79; 95% CI, 0.64-0.98; *P* = .03). The association between trainee participation and the primary outcome, ADR, did not differ by trainee specialty (RR, 1.09; 95% CI, 0.90-1.33; *P* = .38 for gastroenterology vs general surgery trainees). Furthermore, training level was not associated with ADR among gastroenterology trainees (RR, 1.03; 95% CI, 0.91-1.18; *P* = .62 for senior or advanced trainees vs junior trainees) but was associated with ADR among general surgery trainees (RR, 1.12; 95% CI, 1.03-1.34; *P* = .02 for senior or advanced vs junior trainees). However, there was no evidence of effect modification by trainee specialty overall based on the Mantel-Haenszel test for homogeneity, nor was there a significant interaction (RR, 1.14; 95% CI, 0.90-1.44; *P* = .29).

**Table 3.** Univariate and Multivariable Analysis for the Association Between Trainee Participation and Colonoscopy Outcomes

Outcome	Univariate			Multivariable	
	No trainee, %	Trainee, %	*P* value	RR (95% CI)	*P* value
ADR	27.3	26.4	.19	0.97 (0.91-1.03)	.30
ssPDR	5.2	4.4	.009	0.79 (0.64-0.98)	.03
PDR	42.0	39.2	<.001	0.98 (0.93-1.04)	.47
CIR	97.2	96.7	.07	0.93 (0.78-1.10)	.38
Perforation	0.06	0.05	.82	Unavailable^a^	NA

Abbreviations: ADR, adenoma detection rate; CIR, cecal intubation rate; NA, not applicable; PDR, polyp detection rate; RR, risk ratio; ssPDR, sessile serrated polyp detection rate.

^a^ Owing to inadequate event rate for multvariable regression.

## Discussion

In this large population-based cohort study, we found that trainee participation was not associated with most colonoscopy outcome metrics, with the exception of ssPDR. The chance of finding a sessile serrated polyp when trainees were involved was 21% lower than when the procedure was performed by only the attending physician (RR, 0.79; 95% CI, 0.64-0.98; *P* = .03). This finding persisted even after controlling for patient, procedural, and endoscopy covariates and may be explained by the difficulty in detecting these lesions, even by board-certified endoscopists, owing to their sessile or flat morphologic charateristics, indistinct borders, and mucus film obscuring visualization.^18^ This was exemplified by 2 large retrospective cohort studies in which ssPDRs were highly variable between endoscopists, with the most important factor associated with the detection of sessile serrated polyps being the specific endoscopist performing the procedure.^19,20^ Nonetheless, the detection of sessile serrated polyps is highly important given that they have unique clinical and molecular profiles and account for up to one-third of colon cancers.^21^ Ultimately, sessile serrated polyps are difficult to detect endoscopically, and in light of the findings of our study, extra attention should be paid while withdrawing through the right colon when supervising trainees, such as a second look by the attending endoscopist.

Although a lower risk ratio for the ssPDR was associated with trainee involvement, other outcome metrics were not. The ADR, which is directly correlated with the risk of postcolonoscopy colon cancer,^6,7^ and the PDR were not significantly different when trainees were involved and after adjusting for potential confounders. Similarly, prior studies have not found a negative association between trainee participation and the ADR,^22,23,24,25,26^ with the exception of 2 studies that failed to adjust for confounders,^27,28^ 1 small study consisting of only 309 colonoscopies^29^ and 1 study involving an unknown number of gastroenterologists and trainees.^30^ No prior studies examined the ssPDR as an outcome, which is of particular relevance given the difficulty in detecting sessile serrated polyps. Last, in our study, neither the CIR, which is a marker of the ability to technically complete a colonoscopy, nor the perforation rate were associated with trainee participation. The lack of association with CIR was congruent with 2 prior studies that also reported no difference in the CIR when trainees were involved.^23,29^ To our knowledge, no studies prior to ours examined the association between trainee participation and the perforation rate. Although perforation rates were too low in our study for multivariable regression, the crude analysis failed to reveal a difference in this risk when trainees were involved.

To our knowledge, this study is the largest and most definitive to date examining the association between trainee participation and quality of colonoscopy. Prior studies in the field were on a much smaller scale, with our sample size more than 4 times greater than the next largest study.^22,23,24,25,26,27,28,29,30^ Furthermore, prior studies were conducted at single centers,^22,23,24,25,26,27,28,29,30^ involved a small number of trainees and endoscopists,^22,23,24,25,26,27,28,29^ or were restricted to screening colonoscopies.^22,23,27,30^ The end result of these methodological limitations is poor external validity due to the lack of diversity in endoscopy centers, trainees and trainers, and colonoscopy indications. In contrast, our large population-based study involved 71 endoscopists and 55 trainees from both gastroenterology and general surgery, 21 hospital sites inclusive of both academic and community centers, and 35 499 colonoscopies to encompass a wide variety of indications and practice patterns.

Many prior studies^25,27,28^ were also limited methodologically by the lack of adjustment for confounders, which is a basic requirement of observational studies.^14^ In contrast, we used 2 methods to control for confounding: restriction and regression. Restriction prevents the enrollment of individuals who may confound the association between the exposure and the outcome through the use of exclusion criteria to create a more homogenous study population. In our study, the association between trainee participation and quality of colonoscopy may have been confounded by the inherent differences between endoscopists who teach and endoscopists who do not teach residents. Often, these differences may be unmeasurable and impossible to adjust for using statistical methods. By limiting our cohort to only endoscopists who teach trainees, the intangible differences between these 2 groups become irrelevant because the latter were excluded from the analysis. As a result, the study examines only the association between trainee participation and quality metrics among endoscopists who supervise trainees, comparing the performance of endoscopy teachers when trainees are present with the performance of endoscopy teachers when trainees are absent. The disadvantage of this approach is that our results cannot be generalized to endoscopists who do not teach endoscopy given that they were excluded from the study. However, given this group does not work with trainees, the findings of our study would not be relevant to them in any regard. Furthermore, we use multivariable modified Poisson regression to control for the smaller but potentially important differences among the 71 endoscopy teachers captured in our data set. We adjusted for endoscopist, patient, and procedure variables, resulting in a robust estimate of the association between trainee participation and colonoscopy outcomes accordingly.

### Limitations

There are 2 limitations of our study that should be acknowledged. First, although colonoscopy withdrawal time is a quality measure, we did not include it as a study outcome because it was not captured by our data collection form. However, the benefit of a longer withdrawal time is its association with a higher ADR, which is already our study’s primary outcome, and there were no significant differences found.^1^ Second, the precise role of the trainee during the procedure was not captured. In all cases, trainees had hands-on participation and were not merely observers, usually with the consultant in the procedure room, as is routine practice in our region. There is no well-validated and widely accepted tool to measure the extent of trainee involvement during a colonoscopy procedure. Instead, we used training level as a more pragmatic yet clinically meaningful measure because trainee involvement and independence increase with seniority. Ultimately, knowledge of the precise role of the trainee during the procedure is most important when a quality issue is identified to help find the root cause. Because no quality deficit was detected, with the exception of the ssPDR, the lack of data regarding the role of the trainee is largely a moot point. Even for the ssPDR, it would be reasonable to assume that the reduction is due to trainee participation during the withdrawal phase, given this is when the detection of sessile serrated polyps occurs.

## Conclusions

In this cohort study, trainee participation in colonoscopies was associated with a lower ssPDR, although no association was seen with other important quality metrics, such as the ADR, PDR, CIR, or perforation rate. Given that one-third of colon cancers are thought to arise from sessile serrated polyps, the importance of these findings is self-evident. As such, particular attention to the recognition of lesions is necessary, especially in the right colon, when trainees are involved.
